# Shared medication coordination in a social psychiatric residence: adaptation to meet local requirements

**DOI:** 10.1186/s12888-025-06653-2

**Published:** 2025-03-06

**Authors:** Tina Birkeskov Axelsen, Charlotte Arp Sørensen, Anders Lindelof, Mette Spliid Ludvigsen

**Affiliations:** 1 Hospital Pharmacy, Central Denmark Region, Palle Juul-Jensens Boulevard 240, Aarhus N, 8200 Denmark; 2 Regional Psychiatry Randers, Central Denmark Region, Randers, Denmark; 3 Department of Clinical Medicine, Randers Regional Hospital, Aarhus University, Aarhus, Denmark; 4 Faculty of Nursing and Health Sciences, Nord University, Bodø, Norway

**Keywords:** Adaptation, Medication coordination, Shared decision-making, Patient involvement, Social psychiatric residence, Mental health services, Severe mental disorder

## Abstract

**Background:**

Shared medication coordination (MedCo) is vital yet difficult to manage for residents living with severe mental disorders in residential care, where multidisciplinary teams provide support. A successful Shared MedCo model in one residence included three core components: "shared decision-making," "patient involvement” and "MedCo”. This model was effective but transfer to other residential settings needed implementation adaptation. The aim of this study was to meet local MedCo requirements by achieving a good fit between a Shared MedCo intervention core components and a social psychiatric residential context.

**Methods:**

The methodology was guided by a complex intervention adaptation framework involving co-creation with stakeholders to gather iterative feedback. The intervention was adapted through a systematic four-phase process and tested through shared consultations. Ten residents took part in the test, and the intervention’s feasibility and acceptability were assessed.

**Findings:**

The adaptation process ensured a good fit between the intervention’s core components and the new context. Stakeholder input provided crucial content and contextual insights, while planned adaptations laid the foundation for modulating the individual residence Shared MedCo model. Iterative adaptations during the test phase refined the intervention, leading to near-routine performance by the tenth consultation. Residents gained a stronger voice in their healthcare, and all ten had their medication coordinated and optimised. The intervention was found feasible and acceptable.

**Conclusion:**

For effective implementation, complex multidisciplinary Shared MedCo interventions require contextual adaptation and active stakeholder involvement. The shared MedCo intervention offers a guideline for achieving a good fit between the intervention core components and diverse residential contexts, ensuring successful medication coordination for residents living with severe mental disorders.

**Supplementary Information:**

The online version contains supplementary material available at 10.1186/s12888-025-06653-2.

## Background

Severe mental disorders, such as schizophrenia and bipolar disorder, globally affect 64 million individuals [1]. This population accounts for nearly 25% of the global disease burden, exceeding that of cancer and circulatory diseases [2]. It is also characterised by a high multimorbidity prevalence [3], along with elevated hospitalisation and mortality rates [4–8].

Consistent with other studies [9–11], the ‘Global Report on Health Equity for Persons with Disabilities’ (2022) highlights that systemic health inequities, poorer health outcomes and functional limitations contribute to a life expectancy up to 20 years shorter for this population [12], often due to avoidable factors [13, 14]. These factors include higher rates of chronic conditions, untreated diseases [9–11] and fewer physical check-ups and screenings, which result in increased healthcare contacts [15].

Numerous treatment options are available [16] with medical treatment being a primary component [17]. However, medical treatment can be a challenge, and complex medication regimes may appear. Examples are antipsychotics with insufficient or excessive dosing [18, 19], drug interactions between antipsychotic and somatic medications, adverse medication effects [3, 20, 21] and use of multiple antipsychotics without clear benefits [22, 23].

Furthermore, due to multimorbidity and the siloed nature of systems [24–26], multiple healthcare professionals (HCPs), such as general practitioners (GPs) and psychiatrists, may prescribe medications to the same patient, leading to complex polypharmacy. For example, psychiatrists in secondary care may prescribe antipsychotics, which can cause somatic adverse effects like metabolic syndromes (e.g. diabetes) [3], for which conditions GPs in primary care manage the treatment.

When multimorbidity and polypharmacy [27, 28] are present, effective monitoring of medical treatment, e.g. adverse effects and drug interactions, requires medication coordination (MedCo) and shared knowledge among HCPs [29]. However, MedCo is often complicated by the siloed nature of society, which affects healthcare and social living settings, along with collaboration between multidisciplinary, cross-sector HCPs [30]. This may challenge the overall medical treatment, and the task of transferring health information between HCPs often falls on the patient.

Health information transfer can be particularly challenging for patients with significant cognitive disorders who require comprehensive support to manage their lives and maintain treatment coherence. This group of patients may struggle to perceive and process health and medication information independently, and the loss of critical information can negatively impact their medical treatment [9–11, 24]. They often rely on external support; moreover sheltered living arrangements, such as social psychiatric residential settings, may provide an appropriate solution. Residences, staffed by various HCPs and caregiver employees, play a crucial role in patient (resident) care [31]. Residence employees are key to the quality of health care for adults with severe mental disorders and are responsible for supporting residents in managing and coordinating their various healthcare needs [32, 33]. Moreover, they facilitate information exchange between HCPs and residents [32]. However, employees must also navigate a siloed healthcare system while fulfilling their own legal responsibilities [33].

Thus, health coordination and actively involving the resident in their healthcare decisions in practice can be perceived as complex and time-consuming [34]. Consequently, employees may end up speaking on behalf of residents rather than actively involving them in having a voice on their own in healthcare [35, 36]. A solution is needed to provide proper medical treatment for this resident group.

Improvement of treatment communication and interpersonal collaboration [37, 38] and reaching a shared clinical decision between residents and HCPs grounded on the best research evidence and the patient's preferences while respecting their autonomy may be facilitated through a shared decision-making (SDM) approach [39]. Since the initial recommendations for implementing SDM in mental health, research has advanced rapidly over the past two decades [40, 41]. Empirical studies recognise the evidence supporting the impact of interprofessional collaboration through SDM and PI in mental health [42–46]. Existing evidence links SDM to recovery, person-centered care, user engagement, and factors such as ownership, fluctuating capacity, the therapeutic alliance, and changes in clinical attitudes.

In this study, the definition of SDM follows the NICE (National Institute for Health and Care Excellence) definition:

*Shared decision-making is a joint process in which an HCP works together with a person to reach a decision about care. It involves tests and treatments based both on evidence and on the person’s individual preferences, beliefs and values. It makes sure the person understands the risks, benefits and possible consequences of different options through discussion and information sharing* [47].

Furthermore, supporting employees in giving residents a voice [48, 49] and enhancing residents' autonomy and self-determination [35] may be achieved through a patient involvement (PI) approach. Thus, PI is an integral part of SDM, as in the present study. However, due to the target residents' cognitive circumstances, PI requires extra effort, including additional support, such as carer staff support and specialised approaches, to help residents advocate for themselves and have a voice in their own treatment. Thus, in this study, PI adds an extra layer to SDM, requiring attention to both concepts.

Additionally, effective communication and information exchange among multidisciplinary teams, along with proper care coordination, can be ensured through health coordination and MedCo [27, 50].

A Danish residence integrates these core components—SDM, PI and MedCo—through 'Shared Residence Consultations' to ensure SDM, 'supported PI' to promote PI and 'Organised MedCo' to address MedCo needs [51–53].

For over a decade, this ‘*Shared MedCo’* approach has been accepted and practised in this residence. The long-term effects include earlier disease diagnosis, improved and reduced medication use, fewer healthcare contacts, improved quality of life for residents, enhanced employee job satisfaction, time savings for all participants, lower cross-sectoral and interprofessional MedCo expenses, and minimal individual effort resulting in broad benefit for the residents and society [51–53].

The intervention also aligns with Danish political efforts, the context of the present study, aimed at strengthen coherent patient care processes [54], and enhancing the benefits of SDM, PI and MedCo for individuals living with severe mental disorders [24, 55, 56]. However, despite several (unpublished) attempts to optimise complex medical treatments for other residents [57–61] by replicating this Shared MedCo model, transferability to other residential contexts remains insufficient. The lack of transferability success may be addressed by acknowledging that replicating interventions often fails without context adaptation due to contextual and population differences [62]. This study aims to explore how to align the core components of a Shared MedCo intervention with local MedCo requirements to achieve a good fit within a social psychiatric residential context.

## Methods

### Design

#### The first phase of the MRC framework

This study was part of a larger project focusing on Shared MedCo for residents with severe mental disorders living in a social psychiatric residence. This study addresses the first of four phases of the UK Medical Research Council's (MRC) Framework for Developing and Evaluating Complex Interventions in Healthcare (developing or adapting complex interventions) [63, 64]. To ensure alignment between the core components of the Shared MedCo intervention and local MedCo requirements, we developed a MedCo adaptation guideline. Following this guideline, we adapted the intervention to fit within a new social psychiatric residence, drawing inspiration from the original residence. Finally, we examined feasibility and acceptability to assess the intervention's fit in the new residence. This is detailed below.

#### The Shared MedCo adaptation guideline

To systematically guide the adaptation of the Shared MedCo intervention in the present study while maintaining its core components [65] and achieving a good fit between the intervention and the new context [66, 67], we constructed a systematic adaptation guideline.

'Adaptation' refers to "the process of intentionally modifying an intervention without competing with or contradicting its core elements or internal logic" [65] to "achieve a better fit between the intervention and the new context" [66, 67].

This systematic step-by-step *Shared MedCo adaptation Guideline (guideline)* was constructed inspired by the MRC core elements (Context, Programme theory, Stakeholder engagement, Key uncertainties, Refinements, Economic) [63, 64], two steps of the ADAPT guideline: Steps 2 (Plan for and undertake adaptations) and 3 (Plan for and undertake piloting and evaluation) [66, 67], and the eight process phases in the ADAPT-ITT model: (Assessment, Decision, Administration/Adaptation, Production, Topical experts, Integration, Training, and Testing) [65].

This guideline facilitated multi-stakeholder co-creation, involving key field stakeholders such as residents, caregiver employees, HCPs and decision-makers. It aimed to address the needs of the target population through planned preliminary adaptations, the modulation of a context-specific Shared MedCo model, and iterative, responsive adaptations. The study methodology followed four phases of the Shared MedCo adaptation process (Table 1).

**Table 1:**
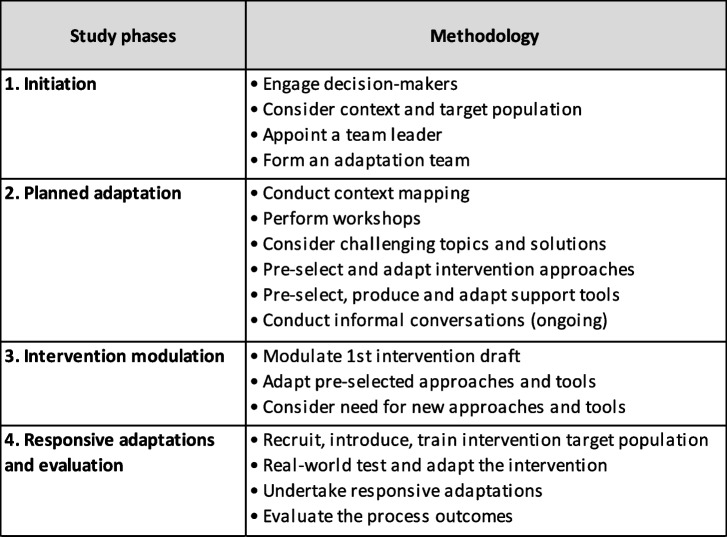
The four-phase Shared MedCo adaptation guideline

Decision-makers were involved from the outset, ensuring that the process was contextually relevant and aligned with the needs of the participants. Collaboration with members of the field was maintained throughout the process.

The principal investigator (investigator) was an experienced clinical pharmacist trained in conducting pharmacist-led residential medication reviews [68, 69] and played a key role in developing and implementing the original intervention. Despite being familiar with the intervention and key stakeholders, neither the investor nor the coauthors had any formal or professional relationships with the target population at the study site during the study.

### Setting and participants

The setting for this study is Danish social psychiatric residences. In Denmark, healthcare is universal, tax-funded and based on the principle of equal access for all citizens [70]. The system is organised across three levels: state, regions and municipalities [70]. The state oversees regulation [71], the five regions manage hospitals and psychiatric care, and the 98 municipalities provide primary care and social psychiatric services [70, 72]. The healthcare system is divided into primary and secondary care sectors, where medication is prescribed by relevant physicians in both [73]. Primary care, such as GP services, manages general care and is available without referral, while secondary care, such as specialised hospital treatment like the somatic and psychiatric hospitals, requires a referral [73]. Healthcare is governed by the Danish Healthcare Act (regulating healthcare and thereby medication) and the Danish Social Services Act (covering non-healthcare services like rehabilitation in, e.g., residences) [71, 72]. Only one shared electronic resource, the Danish *Shared Medication Record*, containing the patient's current medication list, is available for patients, GPs and hospital HCPs, including psychiatrists [74], which challenges cross-sectoral collaboration between HCPs across healthcare sectors and disciplines.

The selected new residential context was a small Danish residence that provided 24-h social psychiatric care for adults with severe mental disorders and somatic illness, primarily assisting residents over 50 with cognitive disorders and a Global Assessment of Functioning Scale (GAF) score mostly under 40, reduced social function and challenges like expressive behaviours and alcohol abuse. GAF is a clinician-rated scale that rates individual persons living with mental disorders for symptom and functioning severity on a scale from 100 (extremely high functioning) to 1 (severe impairment) [75].

This aligned with the original residence that provided 24-h care for adults with severe mental disorders with a GAF score under 40 and somatic illnesses, often with Korsakoff's psychosis, challenging behaviours or legal issues, requiring specialised support for impaired social functioning [51–53].

The selected target population included residents and stakeholders. *Residents* were ten persons (male = 6) between the ages of 55 and 80 living with severe mental disorders, mostly paranoid schizophrenia (*N* = 7) but also bipolar disorder (*N* = 2) and Wernicke-Korsakoff syndrome (*N* = 1) and with a GAF score in the interval 1–40 (1–10 (*N* = 3), 11–20 (*N* = 5), 21–30 (*N* = 0) and 31–40 (*N* = 2)). The residents lived in the ‘new residence’ and received somatic and antipsychotic medications managed by their GP in the primary healthcare sector and a psychiatrist in the secondary healthcare sector. Stakeholders employed: *Residence employees,* which included multiple registered nurses (RN), social and healthcare assistants (some with a professional function of medication responsible (medication carer staff)), occupational and physical therapists, and mental health support workers (collectively: carer staff). *Healthcare professionals,* including two GPs, four psychiatrists, and one regional pharmacist (recruited from a hospital pharmacy) (collectively: HCPs). *Decision-makers* included regional heads from psychiatric and social fields and residence managers. Other *stakeholders* affiliated with the residences were, e.g., administrative staff and clinical pharmacologists.

### Data collection and analysis

This section is structured according to the four-phase Shared MedCo adaptation guideline.

#### Adaptation team

As part of the initiation phase, an adaptation team (team) was carefully and purposefully selected to contribute to co-creative workshops. A skilled residence-employed RN was appointed as a team leader (leader) in agreement with the residence manager. The team was comprised of a seven-member multi-professional group selected in collaboration with the leader, one representative carer staff member and the investigator. Included in the team were explicitly experienced residence context experts: one RN, four medication carer staff, one mental health support worker and one administrative employee.

The team provided context-specific feedback on the intervention's relevance and efficacy purposefully involved all residence staff, contributing insights from daily routines. The leader and the investigator ensured consistency with the core components and the decisions, and the investigator made the final decisions.

#### Planned adaptations

The second phase involved preliminary planned adaptations to prepare for an anticipated good fit between the intervention core components (SDM, PI and MedCo) and the new context.

Initially, a context-specific foundation was established through MedCo-focused context mapping. Over the course of a month, all residence carer staff collectively reviewed and mapped existing medication activities. The team then provided feedback on how to integrate the intervention's core activities ('Shared Residence Consultations', 'Supported PI', 'Organised MedCo') into these existing activities. Furthermore, the adaptation processes were based on input from content experts (the original intervention developers, original residences’ managers and representative staff) according to the content of the original intervention, and insights from other context experts representing the new residential context (residents through their carer staff, HCPs and managers). Naturalistic data and real-world insights into challenges and intervention acceptance were ensured throughout the adaptation process by the leader and the investigator, conducting informal conversations with members of the target population, such as the affiliated HCPs, carer staff and residents [76]. The conversations were facilitated through open, unstructured questions and natural conversation topics that emerged during the dialogue. Findings were documented in field notes and contributed to the adaptation direction within and between the workshops [77]. This approach ensured that the adaptations aligned with the actual needs and perspectives of the target group.

The leader and the investigator initiated the adaptation process by pre-selecting context-specific intervention approaches and supporting tools inspired by those performed in the original residence context [51–53] and supported by existing research evidence. Within the co-creative workshops, the team systematically assessed the pre-selected material and, based on their field experience, anticipated implementation challenges for the individual core activities by considering the target population's needs and preferences. Next, the team suggested new approaches and tools to resolve these challenges. External HCP experts (GPs, psychiatrists, clinical pharmacologists and pharmacists) and content experts were consulted as needed. Context-specific intervention approaches and tools were produced, adapted and prepared to modulate phase three's residence-specific Shared MedCo model. Supplementary file 1 summarises the MedCo adaptation activities,

#### The Shared MedCo intervention modulation

Phase three involved modulating the new context-specific Shared MedCo model.

The 1st draft of the model and its affiliated intervention-supporting tools deemed appropriate for the new target population were modulated by the leader and the investigator based on insights from the earlier adaptation phases and feedback from the team. The ‘*Template for intervention description and replication*’ (TIDieR) inspired the process [78].

The supporting tools were compiled in a toolbox and categorised according to the need for support in the introduction to or performance of the intervention. Members of the target population reviewed adaptations, suggested new adaptations and approved tools relevant to their specific fields and professions, e.g. individual introduction leaflet and work descriptions.

The result of phase three was a residence-specific Shared MedCo model.

#### Responsive adaptations

Finally, in Phase four, a real-world test implementation of the residence-specific Shared MedCo model involving ten residents provided insights into whether and how the new Shared MedCo model could effectively be implemented in the new context.

Residents receiving both somatic and psychiatric treatment were selected by the leader and investigator, and recruited through their carer staff, who were recruited by the leader. Healthcare professionals (GPs, psychiatrists, pharmacists) were recruited by the investigator. The residence manager facilitated contact with the psychiatric decision-maker and supported the recruitment of a GP, who was responsible for most of the selected residents and conducted bi-weekly consultations prior to the study.

An introduction, given high priority [51], was provided by the investigator to the intervention deliverers (HCPs and carer staff). HCPs were introduced individually. Carer staff were introduced during staff meetings. Residents were introduced by carer staff who had been trained by the leader and the investigator.

The three core activities were implemented simultaneously. Iterative, responsive adaptations to the prepared model approaches and tools and new tools were developed in response to unintended consequences and implementation challenges.

Shared residence consultations involving the resident and affiliated HCPs and carer staff were conducted through ten iterative test cycles during the test period. Two residents were seen per day over five days. A minimum of two weeks was introduced between consultation days to give time for the team to implement responsive adaptations.

Newly co-created tools, such as introduction leaflets, PowerPoint presentations and intervention tools, were used and iteratively adapted. Participant training was purposefully conducted, and daily barriers were addressed through a collaborative effort between the leader, investigator and team, with support from the residence manager.

**Fig. 1:**
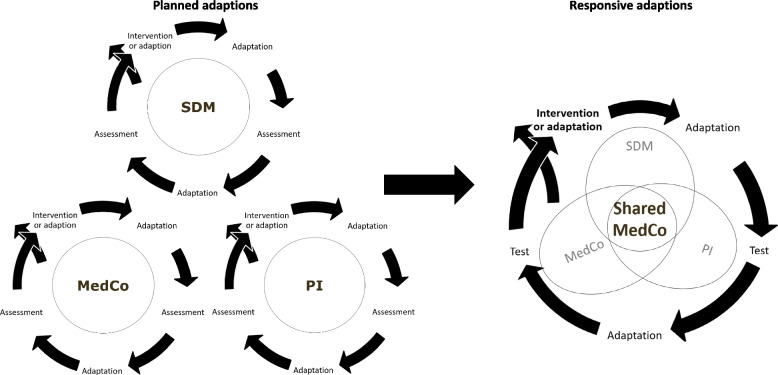
The MedCo adaptation process – from core components to Shared MedCo intervention. Planned adaptations: Individually and iteratively assess intervention core components (SDM, PI, MedCo) for anticipated implementation challenging topics. Responsive adaptations: simultaneously testing core components through ten iteration circles

An overview of the MedCo adaptation process phases 2 and 4 is given in Fig. 1.

#### Feasibility and acceptability evaluation

An initial assessment of the intervention’s feasibility and acceptability for the new target population was conducted through a feasibility and acceptability evaluation.

Feasibility was assessed by evaluating the process outcome of the intervention to gain an early understanding of 'Reach' (contact with the target population), 'Dose' (quantity implemented) and 'Fidelity' (delivered as intended) [79].

Acceptability was assessed by evaluating the intervention’s appropriateness and identifying implementation challenges [80]. This was done by measuring the target population’s (carer staff and HCPs) acceptance of the intervention [67, 79] based on their anticipated or actual responses [81]. Acceptability was evaluated at three stages: prospectively (three months before testing to assess anticipated intervention acceptance), concurrently (during testing to gauge real-time perceived implementation acceptance) and retrospectively (two months after testing to evaluate experienced model acceptance). The evaluation was based on the seven constructs of The Theoretical Framework of Acceptability (TFA)—affective attitude, burden, ethicality, intervention coherence, opportunity costs, perceived effectiveness and self-efficacy [81]. A seven-question Likert scale questionnaire, rated 1 to 10 and based on the TFA constructs [81], was developed (see Supplementary file 2). Carer staff completed the survey during three staff meetings (prospective, current, retrospective), while HCPs did so after their individual introduction (without follow-up). Responses were summarised as percentages based on the mean values across the seven TFA constructs.

#### Medical change

Medical changes prescribed during the SDM process were found by counting individual residents who were prescribed some kind of new medicine treatment.

### Adaptation timeline

**Fig. 2:**
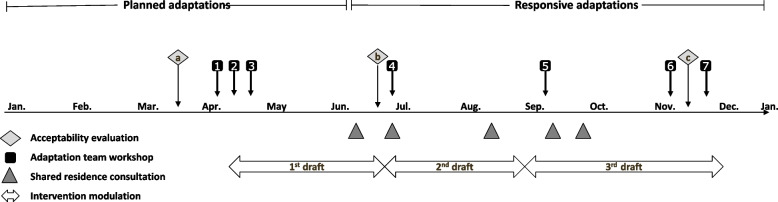
Adaptation timeline. The horizontal arrow represents the timeline for the ongoing adaptations

The 13-month adaptation process (January 2022–January 2023) is illustrated in Fig. 2.

## Findings

This section is structured according to phases two, three and four in the four-phase Shared MedCo adaptation guideline.

### Phase 2: Planned adaptations formed the Shared MedCo intervention

#### Context mapping revealed activities and processes

The initial context mapping revealed existing medication activities. It clarified internal processes, including medication reconciliation and administration, daily communication with residents, collaboration between carer staff and HCPs, legal compliance and documentation.

Based on the context mapping and informal conversations, the team identified key intervention challenges across three critical topics— collaboration, communication and coordination— associated with the individual participant introduction and practical performance of the core activities. Build on these challenges and inspiration from the original residence, the team identified anticipated solutions to address the topics (Table 2).

**Table 2:**
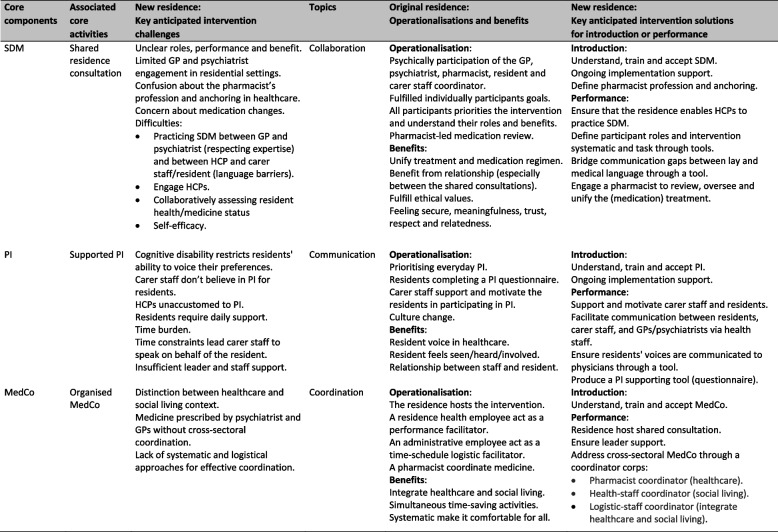
Key anticipated intervention challenge and anticipated practical solutions

*SDM* Shared Decision-Making, *PI* Patient Involvement, *MedCo* Medication Coordination, *GP* General Practitioner

Based on findings from phases one (initiation) and two (planned adaptation), the new context-specific Shared MedCo model was developed by fitting the Shared MedCo intervention core activities with the residence context.

#### Shared MedCo intervention core activities operationalised core components

*Regarding SDM* (shared residence consultation), the original residences' consultation approach was adopted. A pharmacist-led, cross-professional medication review formed the basis for the SDM performed between the GP and the psychiatrist.

*Regarding PI* (Supported PI), the starting point was a PI questionnaire and an affiliated carer staff supporting and motivation approach, which has been routinely practised and iteratively adapted over the past decade in the original residence.

*Regarding MedCo* (Organised MedCo), medication-related tasks performed in advance were consolidated and coordinated concurrently. The original intervention approach, which included simultaneous annual GP health checks, psychiatrist assessments and residence legal responsibilities, was adapted to fit the new residence through three coordinated roles managed by a coordinator corps: 1) A pharmacist coordinator conducted the pharmacist-led medication review and coordination outside the residence context according to the healthcare setting, ensuring that prescriptions from the GP and the psychiatrist were coordinated. 2) A health-staff coordinator managed MedCo within the residence context according to the social living setting, ensuring medication activities and communication with HCPs, carer staff and residents were coordinated. 3) A logistic coordinator handled the timing and scheduling of integrated healthcare and social living MedCo activities, ensuring alignment with the shared residence consultation for all participants.

#### Shared MedCo intervention supporting tools

The supporting introduction and performance tools were iteratively assessed, de-novo developed and/or adapted to align with the three intervention core activities.

The key supporting tools were compiled into a toolbox and categorised according to their role in supporting the introduction and performance of the interventions.

Table 3 lists essential supporting tools, their adaptation phases and process affiliations, as identified through planned and responsive adaptations, and illustrates their association with the three core components (SDM, PI and MedCo).

**Table 3:**
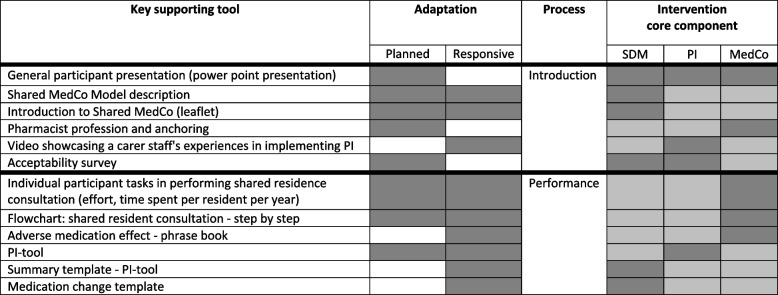
Key supporting tools

The dark grey illustrations in the core component column represent the strongest associations with the respective core components

*SDM* Shared Decision-Making, *PI* Patient Involvement, *MedCo* Medication Coordination

### Phase 3: The residential-specific Shared MedCo model was modulated

The 1st draft of the modulated Shared MedCo model, which was assessed purposefully for the target population in the new residence, is outlined in Supplementary File 3.

**Fig. 3:**
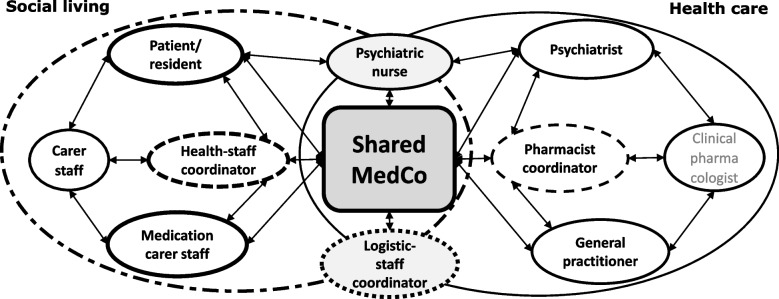
The new residences’ shared consultation: participants and field affiliations. Dashed circles: coordinator roles. Arrows: participants who are involved in person during shared residence consultations and interpersonal relations. Bold text: Participants in the shared residence consultation. MedCo: Medication Coordination. Shared MedCo: representing the shared residence consultation where Shared MedCo is performed. The graphic illustration is inspired by the MIND-IT framework [37, 38]

Figure 3 visualises the complex intervention set-up, illustrating participants in the shared consultations, their interrelations and their field affiliations. Additionally, the figure explains the involvement of multiple stakeholders in the consultations and how the healthcare and social living areas come together in the intervention.

Biweekly consultations were scheduled on Thursdays, aligning with the GP's appointments at the residence. The medication list was unified in a 30-min shared consultation, and treatment plans were aligned through participant medication reasoning, negotiation and agreements.

### Phase 4: Responsive adaptations refined the Shared MedCo model

Testing the first draft of the new shared MedCo model revealed real-world challenges for implementing the latest approaches and their tools. Responsive approach and tool adaptations improved the fit between the core activities and the context. Key responsive challenges, the adaptation team explanation and the corresponding adaptations are detailed in Table 4.

**Table 4:**
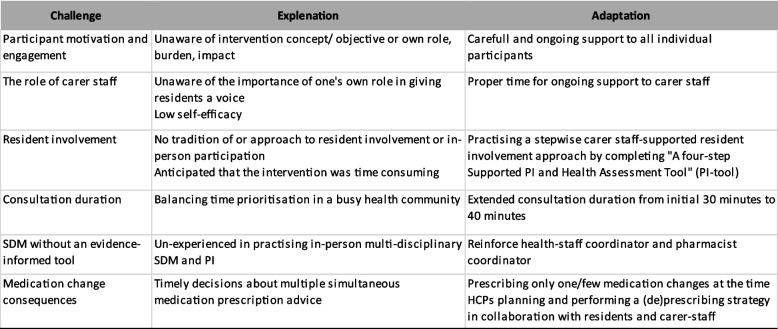
Key responsive adaptations of Shared MedCo to pilot test challenges

*SDM* Shared Decision Making, *PI* Patient involvement, *HCP* Healthcare professionals

During the study period, the residence's Shared MedCo model underwent three draft iterations. The intervention flow diagram is illustrated in Supplementary file 4.

The PI questionnaire adopted from the original context was the most significant responsive adaptation. It underwent 12 modifications to become "The Four-Step Supported PI and Health Assessment Tool" (PI tool). For example, informal conversations revealed that:

*“The PI approach and PI tool should succeed in giving different residents a voice as comprehensively as possible, including those with cognitive disorder and non-verbal communication and ensuring that these insights reached the HCPs.” (carer staff)*

This led to a PI tool with very detailed and concrete symptom descriptions that were helpful for carer staff with backgrounds other than healthcare.

Also, the PI tool was adapted to align medication treatments to be useful as a base for the pharmacist-led medication review and to meet and streamline legal obligations for the GP, psychiatrist and residence.

*“It would be beneficial if all our legal obligations could be carried out simultaneously and incorporated into the PI tool.” (Administrative staff)*

In practice, the four steps were operationalised over a month, with the starting point based on each resident's (patient’s (pt’s)) capabilities. Step 1: "Pt. voice": completed by the resident; Step 2: "Supported pt. voice": carer staff assist the resident; Step 3: "Advocacy pt. voice": carer staff complete any remaining information; Step 4: "Translate pt. voice to health language": the health-staff coordinator summarises and prepares the results for the HCPs.

The PI tools were completed as planned after resolving the initial implementation challenges. Challenges included accepting the approach's initial time-intensive nature, residents' intensive involvement and the practical aspects of logistics and implementation.

The PI tool was the foundation for all ten residents' shared medication negotiations and treatment agreements. The team found the PI approach to be a revelation, as reflected in this carer staff’s quote:

*"I was sceptical at first. I was probably one of those who didn't believe we could involve the residents. But I have to say that I'm wiser now. We CAN involve ALL the residents when we work this way." (Carer staff)*

### Feasible and acceptable Shared MedCo intervention change medication

The *feasibility* evaluation of the Shared MedCo intervention found that by the end of the testing period, the intervention came into contact (reach) with the target population, namely residents (100% according to giving a voice to the residents, 60% according to in-person representation), medication carer staff (90%), GPs (100%), psychiatrists (60%) and pharmacist (100%)). If there was no carer staff representation to support the resident, the medication carer staff took over the task. Table 5 provides an overview of participants' participation and achieved medication changes.

**Table 5:**
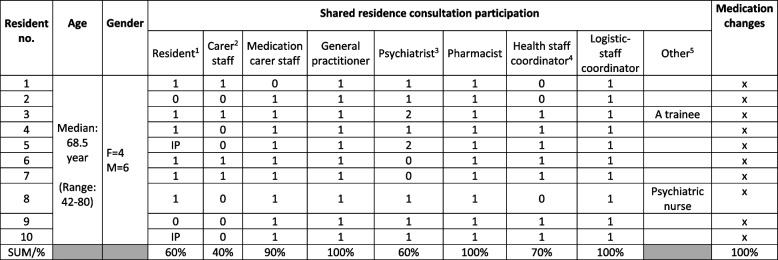
Participants in shared residence consultations and achieved medication changes (*N*=10)

The investor attended all consultations as an observer and intervention supporter, with minimal interruptions

*IP* Impossible participation

^1)^ 1: Resident participated; 0: Resident dropped out immediately before the consultation

^2)^ 1: Carer staff supported the resident; 0: Medication carer staff represented the carer staff

^3)^ 1: Psychiatrist participated; 0: No psychiatrist available during the test period; 2: No psychiatric affiliation during the test period

^4)^ 1: The health-staff coordinator participated; 0: The investigator acted as a stand-in

^5)^ 1: Other external participants involved

During part of the testing period, no permanent psychiatrist was available for the residents, which affected two residents. One resident was referred to a psychiatrist, while the other was not in advance affiliated with a psychiatrist during the test period. Additionally, one resident was hospitalised, and another refused to participate physically but accepted that the carer staff advocated on their behalf as a proxy. On the day of the shared consultation, two residents cancelled their physical participation—one due to anxiety, and the other expected the consultation to take place in their private room. However, due to communication challenges, this arrangement was not implemented as anticipated. For residents who were not physically present (40%), their medication carer staff and the health-staff coordinator spoke on their behalf, based on the PI-tool notes, to ensure that their concerns were communicated to the HCPs. Exposure to the intervention was observed (dose) for all participants, and all ten residents received *medication* reviews and adjustments of their medication regimens. The intervention was assessed and delivered as intended (fidelity).

Following the Shared MedCo adaptation guideline, a good fit between the core activities and the context was achieved, and near-routine implementation, as recognised by the adaptation team, was reached by the end of the testing.

The *acceptability* survey measurements showed participant acceptability above 80%. Prospective acceptability (*n* = 62) was slightly higher (87%) than acceptability during the testing period (concurrent; *n* = 48; 82.3%) and retrospective acceptability (*n* = 41; 82.6%).

The team found that acceptance was highest in the affective attitude and ethicality constructs in the prospective acceptability evaluation. The greatest challenge was self-efficacy among carer staff. Addressing this challenge improved their motivation and confidence, leading to greater participation and contribution during consultations.

Informal conversations with the target population supported the acceptability measures and instilled confidence in intervention development. Conversations indicated that the Shared MedCo intervention was generally considered acceptable, with praise for the residents' involvement and the inclusion of the pharmacist. The following examples reflect the improved acceptance of the Shared MedCo intervention associated with the seven constructs of TFA [81] and illustrate how this approach contributed to the intervention’s confidence and direction.

A resident remarked:

*"I was nervous, but Anna (carer staff) helped me. It felt ok safe talking to both my physicians, and I’m glad that I did. I’ll be more confident next time." (Resident)*

A carer staff added,

*"Although Peter (resident) was extremely upset before the consultation, I calmed and motivated him to participate. Afterwards, he proudly shared his experience with the residence administrative staff. He had never done anything like that before.” (Carer staff)*

These quotes reflect improved acceptance regarding ‘self-efficacy’ and ‘affective attitude’ and give confidence to the PI approach and its feasibility.

HCPs confirmed the intervention's acceptability, stating:

*"It is a considerable quality improvement. The shared consultations work well. However, they are most effective when both the psychiatrist and the pharmacist are present." (GP)*

*"This is not a burden. In the long run, it will not be at the expense of other patients or any other considerations – it will gain time, and it makes sense ... it is something about trust and security." (Psychiatrist)*

These quotes reflect improved acceptance regarding ‘affective attitude’, ‘burden’, ‘opportunity cost’ and ‘perceived effectiveness’ and gave confidence to the SDM and MedCo approach and their feasibility. Furthermore, several GP and psychiatrist pairs confirmed their acceptability by making an agreement for their collaboration for the following year.

An adaptation team member articulated the contribution of the pharmacist:

*"The pharmacist’s role made a lot of sense as the physicians were now making shared decisions, and the medication was actually coordinated. I noticed the difference when the pharmacist was absent after the ten pilot consultations. The interpersonal dynamic shifted and became – more distant." (Adaptation team member)*

The quote reflects ‘intervention coherence’ provided when a pharmacist was involved and gave confidence to the SDM approach and its feasibility.

A decision-maker stated:

*“This intervention - with its holistically treatment and involvement of all residents - speaks directly into my personal values for working with this group.” (Decision-maker)*

This quote reflects improved acceptance regarding the ‘ethical’ dimension of the Shared MedCo intervention and conveys a collective viewpoint shared by all participants.

Overall, establishing Shared MedCo in the new residence resulted in a more resident-centred medical treatment. Several factors characterised the key findings. The intervention was grounded in evidence-based practice and implemented systematically according to the evidence-informed Shared MedCo adaptation guideline. Following the Shared MedCo adaptation guideline, the team focused on integrating core components rather than imposing a top-down intervention.

The residence’s own Shared MedCo model was modulated to fit its specific context, with adequate time and support allocated for implementation. Planned and responsive adaptations were vital to addressing emerging challenges, and the coordination corps was established to ensure smooth execution.

A pharmacist played a supportive role by bridging the work and communication between GPs and psychiatrists, facilitating SDM. Additionally, carer staff were empowered to help residents express their healthcare needs, giving them a stronger voice in the process. The study indicates that the Shared MedCo intervention was both feasible and acceptable and that medication changes were prescribed for all residents. Overall, these elements combined to create a more effective and resident-focused approach to medical treatment within the residence.

## Discussion

This adaptation study successfully provided coordinated medical treatment for residents living with severe mental disorders in a residential setting by achieving a good fit between the Shared MedCo intervention core components and the residential context. The Shared MedCo intervention optimised medicine for all residents by coordinating and facilitating shared decision-making among physicians and providing residents with a voice in their healthcare. Through careful implementation, the intervention was nearly routine practice by the end of the ten real-world testing iterations.

In this section, we discuss the overall findings in relation to existing literature, focusing on two key areas: first, the characteristics anticipated suitable for the Shared MedCo adaptation guideline, and second, the most significant findings related to the three evidence-informed Shared MedCo intervention core components and the study barriers. Finally, we address the study's strengths and limitations and offer clinical recommendations.

### The Shared MedCo adaptation guideline

To overcome past replication challenges with Shared MedCo and ensure a feasible and acceptable implementation, we identified which key properties an adaptation guideline should operationalise to ensure a good fit between the core activities, which define the intervention’s core components, and local requirements.

The resulting guideline was designed as a straightforward, systematic and flexible co-creative four-phase Shared MedCo adaptation process, which was adequate for real-world implementation of Shared MedCo. These characteristics align with existing research. Multidisciplinary approaches emphasise the importance of adaptation guidelines that respond to specific contextual needs, ensuring greater relevance [66, 67], and that real-world implementation and adaptations enhance the evidence base and transferability [35, 82]. Key elements included an evidence-informed and systematic approach grounded in rigorous existing complex intervention and adaptation research evidence [63–67], co-creation and stakeholder involvement [63] and iterative adaptations for contextual integration [63]. Aligning the study with stakeholders' co-creation and real-world needs established a comprehensive and integrated approach and made the intervention relevant and applicable [66, 83]. Therefore, contextual adaptations were essential to achieve a good fit between the Shared MedCo interventions core components and the residential context. Additionally, investing sufficient time and support in developing and practising the four phases of the guideline likely enhanced the model's practicality and future transferability of the evidence-informed Shared MedCo core components [82]. Following ten real-world test iterations, Shared MedCo was nearly fully established. This success was likely attributed to the comprehensive, iterative and collaborative context-specific adaptations guided by the newly developed Shared MedCo adaptation guideline. However, potential barriers to this approach must be acknowledged. Such adaptation processes are often time-intensive burden for both stakeholders and researchers, which may deter new implementers from adopting and committing to the intervention. Nevertheless, leveraging the Shared MedCo adaptation guideline and dedicating the requisite time to foster collaboration and engage field representatives proved to be a worthwhile investment, despite its initial time demands.

### The Shared MedCo intervention Core components

Several coordination findings contributed to fitting the core activities with the context. Among these, the most impactful were the pharmacist’s involvement in Shared residence consultation (Core component: SDM), the PI tool’s integration into Supported PI (Core component: PI) and the establishment of the coordinator corps in Organised MedCo (Core component: MedCo). These key contributions, which were instrumental in delivering the three core components, are further elaborated upon below.

### Core component SDM

Besides coordinating medicine lists, the pharmacist surprisingly played a crucial role in facilitating SDM between GPs and psychiatrists. Rubio-Valera et al. (2014) showed that pharmacists may play diverse roles in mental health care, including working in multidisciplinary teams, managing medication therapy and reducing antipsychotic polypharmacy [84]. Also, a realist review (UK 2023) found that GPs are more likely to accept and discuss medication changes in fragile older people when a pharmacist-led medication review with a clear plan and rationale was presented [85]. These findings, which align with those of the present study underscore the potential for greater pharmacist involvement to enhance physicians’ confidence and willingness to engage in SDM, thereby facilitating the adjustment of complex medication regimens.

### Core component PI

"The Four-Step Supported PI and Health Assessment Tool" (PI tool) enabled the effective capture the residents' voices, even when minimal [48, 49], by tailoring the process to each resident's cognitive abilities and providing comprehensive support through the carer staff [36]. This ensured that residents' voices were communicated to the HCPs, allowing them to be treated on par with other patients. A barrier to the effective implementation of the PI tool was the reduced self-efficacy among carer staff in collaborating with HCPs. For the PI approach to succeed, it is essential to raise carer staff’s awareness of their critical role and provide them with the skills to confidently voice concerns about their residents. Additionally, managers must prioritise empowering carer staff by fostering an environment where their input is valued and heard [49, 86]. By addressing these challenges, the PI tool holds the potential to ensure that all residents, regardless of the severity of their cognitive impairments, have a voice in their healthcare and MedCo.

### Core component MedCo

This study coordinated medicine through SDM and PI. This approach was echoed by a study from the Netherlands (2015), which found that coordinated SDM and PI improved relationships, better aligned decisions with the individual patient’s needs, reduced repeat consultations and increased patient satisfaction [87]. The coordinator corps, comprising a pharmacist coordinator, a health-staff coordinator and a logistic-staff coordinator, overseen by the health-staff coordinator, managed the practical and professional aspects of integrating healthcare and social living in MedCo. In the following, these coordinators are discussed.

Engaging a hospital pharmacist in the role of *pharmacist coordinator* to oversee MedCo across healthcare levels and multiple prescribing physicians enhanced the quality of medication lists and treatment. The involvement of a pharmacist in coordinating medication lists is highlighted in several national and international studies [53, 84, 88–93], including medication management in mental health [84].

The social living MedCo, which involved restructuring residence staff tasks to establish the role of a *health-staff coordinator*, was crucial for aligning multiple participants simultaneously while maintaining a high level of healthcare quality. The role of a nurse health-staff coordinator, recognised internationally, is pivotal in addressing challenges within fragmented healthcare systems. This role enhances coherence between patients and carer staff, facilitates effective communication between HCPs and patients, and ensures that care plans are followed [94, 95]. This coordinator function was deemed essential for the overall success of the intervention. Additionally, and in line with existing research evidence [35], the role of a *logistics coordinator,* responsible for streamlining practical duties and managing coordination on behalf of all participants, was found to relieve other participants from these responsibilities.

While this study demonstrates successful intervention implementation, as in existing evidence [42, 43, 96, 97], several barriers remain. The complexity of the set-up, the involvement of multiple participants and the perception of the intervention as complex, unmanageable, and time-consuming can make such adaptation methods stressful for both the participants and implementers [51]. This burden may potentially deter new implementers from accepting and committing to the intervention, significantly impacting its transferability to other residential settings. On the contrary, the co-creative approach, informed by contextual research findings, may have strengthened the results and reduced implementation challenges and research waste [83, 98]. Additionally, early stakeholder collaboration helped address initial challenges, and, also, adapt the intervention to the specific context, likely improving the intervention’s overall acceptability and feasibility [48, 99–102]. These findings were consistent with those of Hawkins et al., who found that field involvement in the research process could enhance the acceptability of the intervention contents [83]. Also, participants from the original residence viewed these methodological approaches and multi-person participation as beneficial and as a minor personal burden compared to the significant benefits for both individuals and society [51–53].

Furthermore, allocating adequate time for a comprehensive, systematic and co-creative implementation and adaptation approach aligns with Bonde et al. (2018) [103], who found that, despite an evidence-based design, their intervention failed to produce the expected context change. They attributed this to insufficient time and resources in the initial planning and motivation phase [103]. Consequently, Bonde et al. recommended that future implementers allocate adequate time and resources to planning and engaging the target population [103].

In line with the MIND-IT framework, which states that engagement of all involved participants is necessary for the SDM process to precede [37, 38], our study underscores the importance of allocating sufficient time for participant introduction, motivation and contextual adaptations to ensure acceptance from all participants.

We successfully implemented an intervention that unified healthcare obligations and medical treatment, facilitating health information exchange between HCPs and across sectors. This integrated healthcare and social living approach addressed previous MedCo challenges, providing proper medical treatment for a vulnerable population.

### Strengths and limitations

The *strengths* of the new Shared MedCo intervention adaptation approach included the effectiveness of the step-by-step guideline. This guideline offered key adaptation considerations to ensure a good fit between the evidence-informed core components and the new context, ultimately resulting in a feasible and acceptable MedCo intervention capable of delivering proper medication treatment [66, 67]. Another strength was the high degree of co-creation, involving content and contextual knowledge experts from both the original and the new residential context. This collaborative approach provided advanced and contextually relevant insights, enabling targeted adaptions to meet local requirements.

Although the study makes a significant contribution to the establishment of Shared MedCo, several limitations should also be acknowledged. The following will address the key limitations that may have affected both the implementation of the intervention and the transferability of the findings.

A potential *limitation o*f this study was that the investigator, a pharmacist experienced in the field in advance, selected and invited most participants and assessed the intervention's adherence to its core components. This involvement could raise concerns about selection bias, potentially influencing the results. However, close collaboration with the target population and co-authors from psychiatry, pharmacy and nursing areas was used to mitigate these issues and ensure transparency [104, 105]. Additionally, the investigator's expertise did not extend to practical healthcare, social living practice or the general issues in residence contexts.

Another limitation is the small-scale study method. Despite the high level of feasibility and acceptability, this small-scale complex intervention underscores the need for further feasibility studies. Allocating time for initial contextual adaptation in co-creation with the individually experienced residence participants will improve understanding of the acceptability, feasibility and effectiveness of the Shared MedCo intervention before full-scale implementation.

Also, a limitation is the intervention transferability. Although the intervention showed promising results in social psychiatric residential settings, it was specifically developed for this context. Consequently, the findings may not apply to broader populations or other settings. This raises concerns about its transferability to institutions such as hospitals or private care homes, which may differ in organisational structures, resources and patient/resident needs. Further research is required to assess whether the intervention can be adapted and effectively implemented in these alternative settings and to evaluate its impact across different institutional environments.

For sustainability, we recommend investigating the participants' experience with the adaptation approach, the pharmacist’s contribution to SDM and the PI tool in scale. Furthermore, the intervention could benefit from an effectiveness study on a larger scale where the long-term effects on medication, residents’ quality of life, carer staff and HCPs’ job satisfaction and healthcare economics are assessed.

## Conclusion

This study demonstrates that medication coordination can be provided for residents treated under shared practitioner responsibility and living in a social psychiatric residence through the implementation of the complex multidisciplinary intervention, ‘The Shared MedCo intervention’, if the intervention is adapted in a stepwise process that ensures alignment of intervention core components with local requirements. In the long run, the intervention has the potential to help reduce the up to 20-year life expectancy gap for residents with severe mental disorders who are treated by multidisciplinary, cross-sectoral teams.

*The main lessons* of this study emphasised the importance of the adaptation team allocating sufficient time and fostering acceptance and motivation among all participants. Establishing a coordination corps, engaging a pharmacist and involving the target population in a co-creative adaptation process were identified as critical steps. Particularly crucial was providing support to carer staff in amplifying residents’ voices in health and assisting HCPs in improving communication and collaboration.

We *recommend* that future implementers adopt our Shared MedCo adaptation approach and draw on our supporting tools to achieve a good fit between the Shared MedCo intervention and the specific residential context, thereby meeting local MedCo requirements and improving medical coordination for residents in social psychiatric residences.

## Supplementary Information

Supplementary Material 1. Summary of the MedCo adaptation activities.

Supplementary Material 2. Acceptability questionnaire.

Supplementary Material 3. A Description of Shared Medication Coordination in Social Psychiatric Residences.

Supplementary Material 4. The Shared MedCo intervention flow diagram.

## Data Availability

The datasets used and analysed during the present study are available from the corresponding author upon reasonable request.

## Abbreviations

GP: General practitioner
HCPs: Health care professionals
MedCo: Medication coordination
PI: Patient involvement
Pt: Patient
RN: Registered nurse (The Danish 'sygeplejerske')
SDM: Shared decision-making
TFA: Theoretical framework of acceptability
TIDieR: Template for intervention description and replication
Mental health support workers: (The Danish 'pædagog’)
