# Supplementary material for: Shared medication coordination in a social psychiatric residence: adaptation to meet local requirements
Source: BMC Psychiatry. 2025 Mar 6;25:209. doi: 10.1186/s12888-025-06653-2 (PMC11887218; doi:10.1186/s12888-025-06653-2)
Supplement: Supplementary file 2 — Supplementary Material 2. Acceptability questionnaire. [file 12888_2025_6653_MOESM2_ESM.docx]

On a scale rated 1 to 10, mark the response you feel is most consistent with how you are feeling right now.

1: Disagree. 10. Agree.

|  |  | Disagree |  |  |  |  |  |  |  |  | Agree |
| --- | --- | --- | --- | --- | --- | --- | --- | --- | --- | --- | --- |
|  |  | 1 | 2 | 3 | 4 | 5 | 6 | 7 | 8 | 9 | 10 |
| 1. | I worry when I think about start working with Shared MedCo |  |  |  |  |  |  |  |  |  |  |
| 2 | It is going to be a burden for me, having to work with Shared MedCo |  |  |  |  |  |  |  |  |  |  |
| 3 | Shared MedCo is consistent with my personal and morale values! |  |  |  |  |  |  |  |  |  |  |
| 4 | I believe that Shared MedCo is going to create more coherence in the resident’s medicine! |  |  |  |  |  |  |  |  |  |  |
| 5 | There will be more important things for me to do, than spend my time on Shared MedCo |  |  |  |  |  |  |  |  |  |  |
| 6 | I believe that we will succeed establishing Shared MedCo |  |  |  |  |  |  |  |  |  |  |
| 7 | I have doubts about being able to participate in Shared MedCo |  |  |  |  |  |  |  |  |  |  |
| 8 | On a daily basis, I use a lot of unnecessary time handling the resident’s medicine! |  |  |  |  |  |  |  |  |  |  |

The back-translated version of the Danish questionnaire based on the seven constructs of the Theoretical Framework of acceptability (1):

- **Affective Attitude:** How an individual feel about the intervention.
- **Burden:** The perceived amount of effort that is required to participate in the intervention.
- **Ethicality:** The extent to which the intervention has good fit with an individual's value system.
- **Intervention Coherence:** The extent to which the participant understands the intervention and how it works.
- **Opportunity Costs:** The extent to which benefits or values must be given up engaging in the intervention.
- **Perceived Effectiveness:** The extent to which the intervention is perceived as likely to achieve its purpose.
- **Self-efficacy:** The participant's confidence that they can perform the behaviours(s) required to participate in the intervention.

1. Sekhon M, Cartwright M, Francis JJ. Acceptability of healthcare interventions: an overview of reviews and development of a theoretical framework. BMC Health Services Research. 2017;17(1):88.
