# Supplementary material for: Shared medication coordination in a social psychiatric residence: adaptation to meet local requirements
Source: BMC Psychiatry. 2025 Mar 6;25:209. doi: 10.1186/s12888-025-06653-2 (PMC11887218; doi:10.1186/s12888-025-06653-2)
Supplement: Supplementary file 3 — Supplementary Material 3. A Description of Shared Medication Coordination in Social Psychiatric Residences. [file 12888_2025_6653_MOESM3_ESM.docx]

**Supplementary file 11 A Description of ‘*Shared Medication Coordination in Social Psychiatric Residences’* (1)**

| **Item** | **Description** |
| --- | --- |
| **1. BRIEF NAME** | Shared MedCo |
| **2. WHY** | The Shared MedCo intervention is provided to ensure medication coordination (MedCo) for patients living with a severe mental disorder in a social psychiatric residence (residence).  Shared MedCo is challenged by a siloed nature of the healthcare system where general practitioners (GPs) and psychiatrists share treatment responsibility. Examples include psychiatrists (in secondary health care) prescribing antipsychotics for which adverse effects, for example diabetes, the GP (in primary health care) prescribe somatic medicine. Despite the presence of severe antipsychotic metabolic adverse effects and potential interactions between antipsychotics and somatic drugs, there is currently no tradition for collaboration or coordination among the healthcare practitioners (HCPs). This leaves responsibility for healthcare information transfer between HCPs with the patients and/or the residence employee (carer staff), who may lack sufficient resources to transfer this health information.  Health information transfer can be particularly challenging for patients with significant cognitive disorders who require comprehensive support to manage their lives and maintain treatment coherence, and the loss of critical information can negatively impact their medical treatment (2-5). Uncoordinated and inadequate medical treatment contributes to significant healthcare costs and a life expectancy up to 20 years shorter for this population (2, 3).  Improvement of treatment communication and interpersonal collaboration (6, 7) and reaching a shared clinical decision between residents and HCPs grounded on the best research evidence and the patient's preferences while respecting patient autonomy can be facilitated through a shared decision-making (SDM) approach (8). Furthermore, supporting employees in giving residents a voice (9, 10) and enhancing residents' autonomy and self-determination (11) can be achieved through a patient involvement (PI) approach. Additionally, effective communication and information exchange among multidisciplinary teams, along with proper care coordination, can be ensured through health coordination and MedCo (12, 13).  The Shared MedCo intervention is a SDM intervention that provides proper medical treatment for adult living in a residence (residents) through integrated healthcare and social living MedCo. Its long-term purpose is to enhance equality and quality of life, to improve health and optimise health resource consumption and thereby contribute to improved life expectancy for residents treated in a shared responsibility between multiple HCPs; additionally, to contribute to employee and HCPs job-satisfaction.  The intervention is a complex multi-stakeholder intervention based on the Medical Research Council’s (MRC) framework for complex interventions (CI) (14, 15). The intervention achieves the essential elements of SDM stated by the MIND-IT framework (6, 7) and is a real-life routine intervention (16, 17).  The intervention is developed in a co-creative implementation and adaption approach between field representants and researchers (18). |
| **3. WHAT – MATERIALS** | The shared MedCo intervention consist of an adaptation approach where three evidence-informed intervention core components, deemed essential for achieving positive long-term results (19), are adapted to achieve a good fit with the residential context.  The three shared MedCo intervention’s core components are: 1) shared decision making (SDM), 2) patient/resident involvement (PI) and 3) medication coordination (MedCo).  The core components are unfolded below.  The shared MedCo intervention implementation and adaption process is guided by a four-phase ‘*Shared MedCo adaptation guideline’* and a *Shared MedCo toolbox* containing multiple supporting tools adaptable for the residential context. |
| **4. WHAT – PROCEDURES** | The procedure of the shared MedCo intervention goes through the intervention core components which are operationalised through three affiliated core activities, e.g., SDM is operationalised through 'Shared Residence Consultations', PI is operationalised through 'Supported PI' and MedCo is operationalised through 'Organised MedCo'.  Two adaption steps, provided in a co-creation approach between decision-makers and field representants leads to a residential modulated Shared MedCo model suitable to the current residence and participants in both the healthcare setting (HCPs) and the social living setting (residents and residence employees).  Planned and responsive iterative adaptations to the model and needed supporting tools are undertaken in response to anticipated and unintended consequences and to implementation challenges. First, the three core activities are adapted separately to achieve at good fit with the context based on anticipated challenges, needs and solutions. Second, the adapted core activities are implemented simultaneously and adapted to achieve a good fit with the context based on real-world implementation. |
| **5. WHO PROVIDED** | In the healthcare setting, participants are:   - A GP: taking care of somatic medicine - A psychiatrist: taking care of antipsychotic medicine - A pharmacist coordinator: conducting pharmacist-led medication review ensuring coordination of somatic medicine and antipsychotic medicine, additionally facilitating SDM communication.   In the social living setting participants are:   - A health-staff coordinator: a residence carer staff with a health background facilitates the health MedCo activities within the residence along with health activities in collaboration with the HCPs outside the residence. A nurse is recommended for this position - A logistic-staff coordinator: a logistic residence employee handles the timing and scheduling, ensuring alignment with the shared residence consultation for all participants. An administrative employee is recommended for this position - A medication carer staff: a medication management support employee facilitates and ensures the activities and responsibilities in the current residents' residence section - 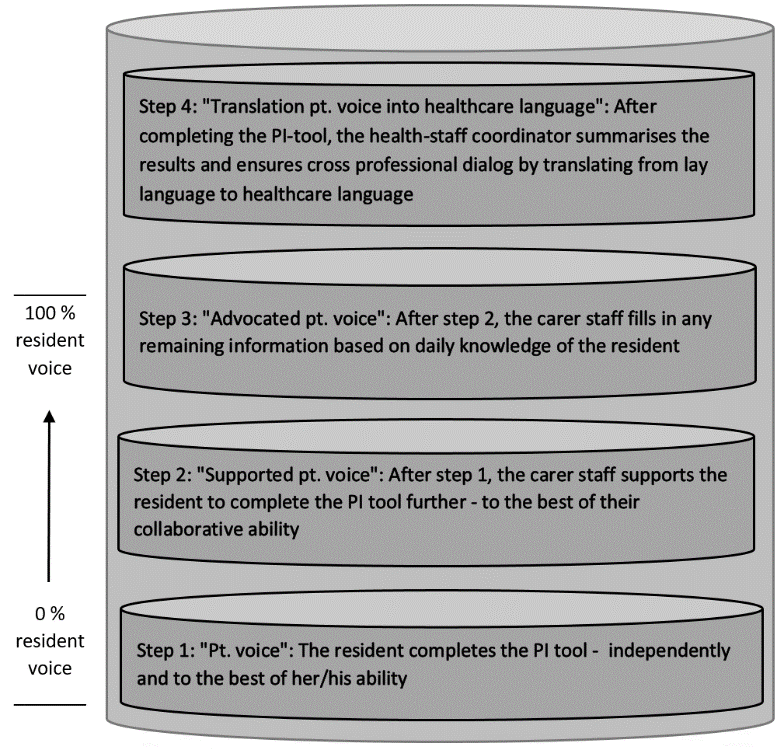A carer staff: an employee (often a mental health support worker) who is entrusted with the task of motivating and practically supporting the resident in the Shared MedCo process. - A resident: a person living with severe mental disorder and treated in shared responsibility between a GP and a psychiatrist. The resident completes a "PI tool" and undergoes healthcare check (supported).   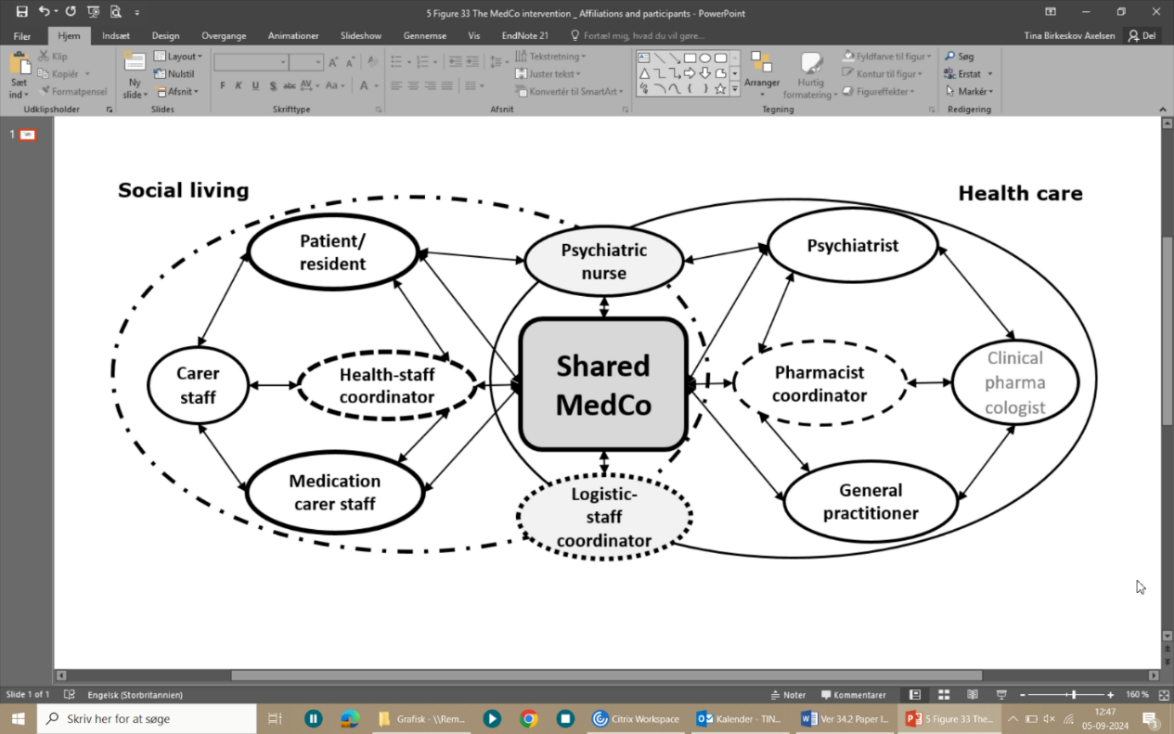The figure shows the residences’ shared consultation participants and field affiliations.  *Dashed circles: coordinator roles. Arrows: Participants who are involved in person during shared residence consultations and interpersonal relations. Bold text: Participants in the shared residence consultation. MedCo: Medication Coordination. Shared MedCo: Representing the shared residence consultation where Shared MedCo is performed.*  *The graphical illustration is inspired by the MIND-IT framework (6, 7).* |
| **6. HOW** | The intervention preparation is contextualised for multiple participants, while the shared consultation is specifically adapted to the need for HCPs and the resident (to ensure resident participation) in the performing SDM. For details see below. |
| **7. WHERE** | The Shared MedCo intervention, preparing and following up on activities, is located in the context of the healthcare and social living participant. Shared residence consultations are located in the residence. |
| **8. WHEN & HOW MUCH** | The shared consultation is suggested annually at the time suggested by the logistics coordinator. Each consultation is suggested to last initially 30 minutes (context adaptable).   - At the beginning of the year, the logistics staff coordinator coordinates the annual shared consultations for all the residents and affiliated participants. The consultation is distributed over the year. - Two months before the shared consultation, the health-staff coordinator initially notifies the HCPs and the medication carer staff. The medication care staff notify the resident and affiliated carer staff and arrange resident health checks. The carer medication staff facilitate, in collaboration with multiple carer staff, support to the resident in completing the PI tool. - One month before; the GP performs a healthcare check. - Two weeks before, the health-staff coordinator sends the completed PI tool to the pharmacist who consults the hospital's "*patient shared medication card*" for medication treatment, biochemistry and anamnesis information and conducts pharmacist-led medication review and coordination. - One week before, the pharmacist sends the results of the medication review electronically to the HCPs. In addition, the health staff coordinator receives the results. - A few days before, the HCPs prepare the consultation (e.g. the medication review), the logistics-staff coordinator prepares the consultation location and catering, and the health-staff coordinator compiles healthcare issues to support and ensure the resident's voice in the negotiation during the shared consultation. - Ongoing; the carer staff (often mental health support workers) facilitates residents' mental preparing, training and motivation. - On the day of the shared consultation, carer staff prepare, motivate and support the resident and ensure that the resident is psychically and mentally prepared to participate. Medication carer staff and carer staff purposefully supplement the resident’s voice. |
| **9. TAILORING/ ADAPTATION** | Research shows that lack of transferability success may be addressed by acknowledging that replicating interventions often fail without context adaptation due to contextual and population differences (20) and that adapting the intervention to fit with the current context is important (18).  Therefore, spending a comprehensive amount of time on co-creative implementation and adaption activities is recommended. Also, it is recommended to adapt and refine the overall intervention approach and the relevant supporting tools to the individual residential context and residents. |
| **10. ADAPTATION** | Experience from a research-based real-world implementation test and adaptation of the Shared MedCo intervention showed that central adaptations were: Supporting participant and carer staff motivation and engagement, the role of carer staff, resident involvement, consultation duration, SDM without an evidence-informed tool, medication changes consequences if more than one or a few changes at the time. |

**The share MedCo intervention core components (SDM, PI, MedCo)**

| ***Core component*** | **Activities, participants and evidence-informed rationale** |
| --- | --- |
| Shared Decision Making  (SDM) | **SDM is performed during the shared residence consultation with preparing activities before and subsequent follow-up activities.**  SDM is recommended as an appropriate model for decision-making in preference-sensitive decisions (21). The approach is essential for promoting patient autonomy and satisfaction, and patients express a desire to participate in SDM (22, 23).  Improvement of treatment communication and interpersonal collaboration (6, 7) and reaching a shared clinical decision between residents and HCPs grounded on the best research evidence and the patient's preferences while respecting patient autonomy may be facilitated through a shared decision-making (SDM) approach (8).  Although SDM involves a slight increase in consultation time, it can lead to decisions that are better suited to the individual patient, improve the HCP-patient relationship, reduce the number of repeat consultations and requests for second opinions, and, in the long term, lead to better treatment adherence and greater patient satisfaction (21). SDM can have a positive influence on whether patients take their medicine as prescribed (24). Engaging in shared, personalised and real-time HCPs reasoning, negotiations and agreements - with the active introduction and participation of all involved participants, including the resident (6, 7) can support a coordinated, comprehensive understanding and health treatment knowledge, including improving MedCo.  This will enhance the SDM process (6, 7) and coherence between the healthcare area and the social living area (25). Furthermore, patients express a desire to be engaged in SDM, which is essential to promote patient autonomy and satisfaction (22, 23, 26).  Although SDM is executed in practice during the shared residence consultation, the process is an ongoing approach, starting with the preparations and continuing with the subsequent follow-ups.  SDM is operationalised with inspiration from ‘*Making Informed Decisions Individually and Together framework’* (MIND-IT) for developing multiple stakeholder interventions in healthcare (6, 7) .  To make shared decisions between stakeholders, we need to support individuals' reasoning to understand the other stakeholder(s) perspectives and support the process enabling the sharing of knowledge, preferences and care planning. To have a good, shared collaboration/decision-making process, multiple factors had to be fulfilled.  All human beings have a fundamental and essential tendency to move towards growth. To facilitate growth in a good direction for the better, human beings had to be motivated to make those changes. Intrinsic motivation promotes behaviour for its own sake and is the most potent motivation factor. Three CORE NEEDS are essential for fostering intrinsic motivation according to "The self-determination theory" (SDT). The three core needs are: (mechanism); *Competence* (the feeling of doing a good job), *Autonomy* (the feeling of having control) and *Relatedness* (the feeling of having good relations) (11).  A good shared decision-making process is supported by all participants making informed decisions individually in their own lives, together, and in a social and healthcare system. The challenge is to make things explicit and implement the final choice in a shared agreement between all participants.  All participants entering a shared decision-making process make their health and social care decisions based on their "life package". MIND-IT illustrates that each stakeholder has their own goal for their context, with their own Experience, Skills, Knowledge, Motivation, Views and Culture.  The content of these "life package" elements differs according to the individual participant, but the "life package" elements are overall fundamental elements which are the same for all participants.  When making decisions, individuals base their reasoning on their own life experiences, roles, and goals within the (SDM) process. For successful decision-making and to avoid conflicts, participants must (6, 7, 11):   - Feel satisfied by meeting their core needs, as defined by the self-determination theory (autonomy, competence, and relatedness). - Recognise that each participant brings a unique 'life package' with different focuses and intervention needs. - Understand both their own needs and perspectives as well as those of others, fostering mutual understanding and effective collaboration.   For example, practitioners base their treatment recommendations on research evidence, while patients make decisions based on their life experiences and daily needs. Effective shared decision-making requires that participants (6, 7):  ⦁ Understand their own reasoning process from problem identification to decision-making (e.g., 'Why am I giving this advice or making this decision?'), allowing them to articulate it clearly to others.  ⦁ Appreciate the other participants' reasoning from problem identification to decision-making. This involves seeing the situation from the other person's perspective, understanding their motives, and taking their reasoning into account to refine one’s own decisions. |
| Patient Involvement (PI) | **PI is performed before, during and after the shared residence consultation**.  PI emphasizes the patient's rights and ability to influence and engage in decisions about their own care (27). PI in health is a priority both in Denmark and globally (28). PI in MedCo, through a shared approach to medication schedules, is seen as particularly positive and valuable for both patients and HCPs (29). Furthermore, the patient's collaboration with a GP or psychiatrist has been shown to optimise complex medical treatment regimens (30-32) and improve communication, resulting in fewer misunderstandings (33).  Patient involvement (PI) emphasizes the importance and right of patients to influence and actively participate in decisions about their care, enhancing autonomy, social confidence and decision-making skills according to their capacity (11, 27).  Collaboration between patients and HCPs, such as GPs or psychiatrists, optimises complex treatment regimens, improves communication and reduces misunderstandings (30-32) (33). Direct involvement of residents in their healthcare decisions, with support from carer staff giving residents a voice (9, 10) ensures a more accurate reflection of their situation and reduces reliance on staff to speak on their behalf. Furthermore, enhancing residents' autonomy and self-determination (11) may be achieved through a patient involvement (PI) approach.  In Shared MedCo, SDM is especially valuable for both patients and HCPs (29), and PI remains a priority in healthcare, both in Denmark and globally (28).  The PI-tool helps residents with cognitive disorders, including those with non-verbal communication, express their preferences and ensure these insights reach HCPs. It also facilitates alignment of medication treatments and simplifies legal obligations for GPs, psychiatrists, and care facilities  The completed PI-tool serves as the foundation for the pharmacist-led medication review and for the shared medication negotiations and treatment agreements in the consultations.  In practice, the four steps were operationalised over a month, with the starting point based on each resident's individual capabilities. Allocating a substantial amount of time (one month) for caregivers to motivate, collaborate with and "read" the resident, led to the completion of PI tools.  The four steps are: Step 1: "Pt. voice": Residents complete the PI tool independently and to the best of their ability. Step 2: "Supported pt. voice": After step 1, the carer staff support the resident in further completing the PI tool to the best of their collaborative ability. Step 3: "Advocacy pt. voice": After step 2, the Carer staff fill in any remaining information based on the daily knowledge of the resident. Step 4: "Translation pt. voice to health language" ensured by the health-staff coordinator. |
| ***Medication Coordination***  ***(MedCo)*** | **Medication is coordinated in a shared in-person residence consultation where healthcare and social living MedCo activities are integrated through SDM and PI.**  Effective communication and information exchange among multidisciplinary teams, along with proper care coordination, can be ensured through health coordination and MedCo (12, 13).  Systematic monitoring and coordinating previously separate medication activities through cross-sectoral and cross-professional coordination and overview of almost identical annual health and medication activities are inherently a time-saver for residents and carer staff. An organised coordination approach, including both approaches and medication, is logical and time-saving.  The fragmented nature of the Danish healthcare system necessitates a dual MedCo approach - both fragmented and collaborative. MedCo is simultaneously carried out in the healthcare area and the social living area, with subsequent integration (17, 34).  **Healthcare area:**  Medication is coordinated in the healthcare setting (outside the residence).  Participants and approach:   - GP: Responsible for resident treatment in the somatic field.   Responsible for somatic healthcare. Prepares reasoning about somatic health and medication.   - Psychiatrist: Responsible for the resident treatment in the psychiatric field.   Responsible for psychiatric healthcare. Prepares reasoning about psychiatric health and medication.   - Psychiatric nurse: Affiliated with the psychiatrist. Maintains regular contact with the resident. Uncovers the residents’ psychiatric condition and informs the psychiatrist. - Pharmacist coordinator: External party to the SDM process. Conducts pharmacist-led medication review (35) and facilitates the coordination of medications prescribed by GP and psychiatrist through a cross-professional pharmacist-led medication review.   Pharmacist-led medication review improves medical treatment (29, 36-40), saves cost (38) and is well accepted (41).   - Clinical pharmacologist**:**   External party to the SDM process. Provides purposeful pharmacological advice in exceptional, complex long-term resident healthcare and medication treatment.  **Social living area:**  Medication is coordinated in the social living setting (inside the residence).  The residence already facilitates annual healthcare contacts, including GP appointments (42, 43), psychiatrist consultations (44), and its own healthcare responsibilities (45).  Consolidate medication-related tasks to be conducted concurrently.  Participants and approach:   - Resident*:* A person living with a severe mental disorder in a residence, receiving both somatic and antipsychotic medications managed by a GP in the primary sector and a psychiatrist in the secondary sector.   Involved in own health decisions and MedCo.   - Health-staff coordinator: Coordinates residential medication activity (medication reconciliation, dialog with the resident and the carer staff). Manages and facilitates communication with HCPs and manages collaboration with the pharmacist.   Facilitates the SDM and PI process before and during the consultations.   - Medication carer staff: A carer staff authorised to manage medication on behalf of the resident. Responsible for and supporting the resident's MedCo and treatment. Ensures the resident voice is heard before, during and after the consultation. - Carer staff: A familiar carer staff*:* nurse (RN), social and healthcare assistants, occupational and physiotherapist, mental health support worker. Offers the resident purposive *ad hoc* support based on knowledge of practical and/or mental needs. Motivates and follows the resident before, during and after the consultation. - Logistic-staff coordinator: An administrative staff. Manages all practical coordination approaches for all participants. Coordinates time-schedule, location and catering. Manages resident flow on the day for the consultations in collaboration with the current residents' carer staff.  **Integrated healthcare and social living areas**  Medication is coordinated through integration of MedCo performed in the healthcare and social living areas.  Participants and approach:  Healthcare and social living MedCo activities are integrated in the shared in-person residence MedCo consultation through SDM and PI (17, 34).  A shared medication list was ensured through a 30 minutes' shared residence consultation based on the pharmacist-led medication review. Shared medication reasoning, negotiation and agreement between all consultation participants were performed facilitated by the health-staff coordinator.  A shared dynamic is reached through practicing and adaptations. |

**References**

1. Hoffmann TC, Glasziou PP, Boutron I, Milne R, Perera R, Moher D, et al. Better reporting of interventions: template for intervention description and replication (TIDieR) checklist and guide. Bmj. 2014;348:g1687.

2. Wahlbeck K, Westman J, Nordentoft M, Gissler M, Laursen TM. Outcomes of Nordic mental health systems: life expectancy of patients with mental disorders. Br J Psychiatry. 2011;199(6):453-8.

3. Laursen TM, Nordentoft M, Mortensen PB. Excess early mortality in schizophrenia. Annu Rev Clin Psychol. 2014;10:425-48.

4. Chan JKN, Correll CU, Wong CSM, Chu RST, Fung VSC, Wong GHS, et al. Life expectancy and years of potential life lost in people with mental disorders: a systematic review and meta-analysis. EClinicalMedicine. 2023;65:102294.

5. Sundhedsstyrelsen. Bedre mental sundhed og en styrket indsats til mennesker med psykiske lidelser. 2022. p. 208.

6. Brown EA, Bekker HL, Davison SN, Koffman J, Schell JO. Supportive Care: Communication Strategies to Improve Cultural Competence in Shared Decision Making. Clinical Journal of the American Society of Nephrology. 2016;11(10):1902-8.

7. Breckenridge K, Bekker HL, Gibbons E, van der Veer SN, Abbott D, Briançon S, et al. How to routinely collect data on patient-reported outcome and experience measures in renal registries in Europe: an expert consensus meeting. Nephrol Dial Transplant. 2015;30(10):1605-14.

8. Elwyn G, Laitner S, Coulter A, Walker E, Watson P, Thomson R. Implementing shared decision making in the NHS. BMJ. 2010;341:c5146.

9. Bovaird T, Loeffler E. We're all in this together: User and community co-production of public outcomes. 2014.

10. Kepplinger A, Braun A, Fringer A, Roes M. Opportunities to address employee voice in health care providers: A scoping review2023.

11. Gagné M, Deci EL. Self‐determination theory and work motivation. Journal of Organizational behavior. 2005;26(4):331-62.

12. Schultz E, McDonald K. What is care coordination? International Journal of Care Coordination. 2014;17:5-24.

13. Möckli N, Simon M, Denhaerynck K, Trutschel D, Martins T, Meyer-Massetti C, et al. How external and agency characteristics are related to coordination in homecare – findings of the national multicenter, cross-sectional SPOTnat study. BMC Health Services Research. 2024;24(1):367.

14. Skivington K, Matthews L, Simpson SA, Craig P, Baird J, Blazeby JM, et al. A new framework for developing and evaluating complex interventions: update of Medical Research Council guidance. BMJ. 2021;374:n2061.

15. Skivington K, Matthews L, Simpson SA, Craig P, Baird J, Blazeby JM, et al. Framework for the development and evaluation of complex interventions: gap analysis, workshop and consultation-informed update. Health Technol Assess. 2021;25(57):1-132.

16. Videbech P BL, Gerdes U, Frederiksen J. Tangkærundersøgelsen. Central Region Denmark; 2008.

17. Specialområde SV. Sundhedstjek 2011 - 2014. Central Denmark Region; 2015 January 1. 2024.

18. Moore G, Campbell M, Copeland L, Craig P, Movsisyan A, Hoddinott P, et al. Adapting interventions to new contexts—the ADAPT guidance. bmj. 2021;374.

19. Axelsen TB; Sørensen CA LA, Ludvigsen MS. Acceptability of shared medication coordination in social psychiatric residence consultations: A qualitative interview study. Manuscript submitted to BMC Psychiatry, submitted 23/6 2024. BMC psychiatry. 2024.

20. Huebschmann AG, Leavitt IM, Glasgow RE. Making health research matter: a call to increase attention to external validity. Annual review of public health. 2019;40(1):45-63.

21. Stiggelbout AM, Pieterse AH, De Haes JCJM. Shared decision making: Concepts, evidence, and practice. Patient Education and Counseling. 2015;98(10):1172-9.

22. Shay LA, Lafata JE. Where is the evidence? A systematic review of shared decision making and patient outcomes. Med Decis Making. 2015;35(1):114-31.

23. velfærd VDnf-oaf. Patienters oplevelse af Collaborative Care 2018 [cited 2024 9/1]. Available from: <https://www.vive.dk/media/pure/yze12ovk/2304951>.

24. Stiggelbout AM, Van der Weijden T, De Wit MP, Frosch D, Légaré F, Montori VM, et al. Shared decision making: really putting patients at the centre of healthcare. Bmj. 2012;344.

25. Voorberg WH, Bekkers VJ, Tummers LG. A systematic review of co-creation and co-production: Embarking on the social innovation journey. Public management review. 2015;17(9):1333-57.

26. Sawan MJ, Jeon YH, Hilmer SN, Chen TF. Perspectives of residents on shared decision making in medication management: A qualitative study. International Psychogeriatrics. 2022;34(10):929-39.

27. Castro EM, Van Regenmortel T, Vanhaecht K, Sermeus W, Van Hecke A. Patient empowerment, patient participation and patient-centeredness in hospital care: A concept analysis based on a literature review. Patient education and counseling. 2016;99(12):1923-39.

28. WHO WHO. Patients for patient safety Unknown [cited 2024 9/1]. Available from: <https://www.who.int/initiatives/patients-for-patient-safety>.

29. Ladegaard N. Tvaerfaglig medicingennemgang - et tilbud i Region Midtjylland til borgere, der er i behandling med antipsykotisk medicin. 2018.

30. Vahdat S, Hamzehgardeshi L, Hessam S, Hamzehgardeshi Z. Patient involvement in health care decision making: a review. Iran Red Crescent Med J. 2014;16(1):e12454.

31. Jayaram G, Doyle D, Steinwachs D, Samuels J. Identifying and reducing medication errors in psychiatry: creating a culture of safety through the use of an adverse event reporting mechanism. J Psychiatr Pract. 2011;17(2):81-8.

32. Huiskes VJB, van den Ende CHM, Kruijtbosch M, Ensing HT, Meijs M, Meijs VMM, et al. Effectiveness of medication review on the number of drug-related problems in patients visiting the outpatient cardiology clinic: A randomized controlled trial. Br J Clin Pharmacol. 2020;86(1):50-61.

33. Howland RH. The challenges of clinical psychopharmacological management. J Psychosoc Nurs Ment Health Serv. 2012;50(5):17-20.

34. Socialpsykiatri. SAMSKABELSE OM BORGERENS SUNDHED Praksisfortællinger fra socialpsykiatrien. Central Region Denmark; 2018 8/1-2024.

35. Kjeldsen LJ, Axelsen TB, Grønkjær LS, Nielsen TR, Tomsen DV, Væver TJ. [Development of patient-specific issues in relation to medicines is a challenge to all health-care professionals]. Ugeskr Laeger. 2014;176(24).

36. Flanagan PS, Barns A. Current perspectives on pharmacist home visits: do we keep reinventing the wheel? Integr Pharm Res Pract. 2018;7:141-59.

37. Murphy K, Coombes I, Moudgil V, Patterson S, Wheeler A. Clozapine and concomitant medications: Assessing the completeness and accuracy of medication records for people prescribed clozapine under shared care arrangements. J Eval Clin Pract. 2017;23(6):1164-72.

38. Cobb CD. Optimizing medication use with a pharmacist-provided comprehensive medication management service for patients with psychiatric disorders. Pharmacotherapy. 2014;34(12):1336-40.

39. O'Dwyer M, Meštrović A, Henman M. Pharmacists' medicines-related interventions for people with intellectual disabilities: a narrative review. Int J Clin Pharm. 2015;37(4):566-78.

40. Aharaz A, Rasmussen JH, McNulty H, Cyron A, Fabricius PK, Bengaard AK, et al. A Collaborative Deprescribing Intervention in a Subacute Medical Outpatient Clinic: A Pilot Randomized Controlled Trial. Metabolites. 2021;11(4).

41. Sundhedsstyrelsen. EVALUERING AF REGIONALE TVÆRFAGLIGE TEAMS VEDRØRENDE MEDICINERING I PSYKIATRIEN 2019 [cited 2024 9/1]. Available from: <https://www.sst.dk/-/media/udgivelser/2020/evaluering-af-tvaerfaglige-medicinteams-i-psykiatrien>.

42. DanskeRegioner. Regionernes Lønnings- og Takstnævn. Aftale om almen praksis : [OK22]. . REGIONERNES LØNNINGS- OG TAKSTNÆVN; 2021.

43. Sundhedstjek R. Almen Praksis - Sundhedstjek for borgere på botilbud. 2022.

44. Medicingennemgang i psykiatrien, fællespsykiatrisk retningslinje [Internet]. . 2024. Available from: <https://e-dok.rm.dk/edok/Admin/GUI.nsf/Desktop.html?open&openlink=https://e-dok.rm.dk/edok/enduser/portal.nsf/Main.html?open&unid=XE3F41FB6A98015BFC1257B8A002872EF&level=OHA&dbpath=/edok/editor/7016.nsf/&windowwidth=1100&windowheight=600&windowtitle=S%F8g>.

45. Sundhedsaftalen. DE 12 SYGEPLEJEFAGLIGE PROBLEMOMRÅDER. 2022.
