# Supplementary material for: Shared medication coordination in a social psychiatric residence: adaptation to meet local requirements
Source: BMC Psychiatry. 2025 Mar 6;25:209. doi: 10.1186/s12888-025-06653-2 (PMC11887218; doi:10.1186/s12888-025-06653-2)
Supplement: Supplementary file 4 — Supplementary Material 4. The Shared MedCo intervention flow diagram. [file 12888_2025_6653_MOESM4_ESM.docx]

3. Inclusion of staff

Healthcare-staff delivers the "*PI tool"* to the carer staff responsible for the resident’s medicine (medication carer staff) and secures the resident’s consent and involvement

**2. Preparation**

One health-staff informs the HCPs and the carer staff about the upcoming shared consultation and initiates *"The four-steps Supported patient involvement (PI) and Health Assessment Tool" (PI-tool)*

**1. Coordination of key participants**

A logistic-staff coordinates dates for the shared consultations between general practitioner (GP), psychiatrist, pharmacist, and relevant staff (annual wheel)

**4. Resident preparation**

The medicine carer staff prepares the shared consultation in collaboration with other carer staff supporting the resident, is responsible for completing the PI tool, make GP appointment for a health care check, and helps the resident with the execution of this as well as the blood sampling

9. Shared residence consultation

The health-staff ensures that the shared consultation is carried out for every participant physically at the residence and that carer staff ensures resident participation (if possible). Logistic-staff manages the meeting room, catering and facilitates the resident flow sequence when necessary. The GP and psychiatrist create in collaboration a shared treatment plan involving reasoning and negotiation with the pharmacist, carer staff and the resident

**5. Health care check**

GP orders blood samples and perform health care check after being notified by health-staff

**6. Acquisition of resident data**

Health-staff sends the completed "PI tool" to the clinical pharmacist

**7. Medication review**

Clinical pharmacist performs pharmacist-led medication review covering both somatic, psychiatric and the cross area between. Results are noted to a psychiatrist in the hospital’s electronic patient record and is sent to the GP and the health-care staff

**12. Follow-up**

Medication carer staff and carer staff follow-up on treatment plan and contacts the GP or the psychiatrist if unexpected events occur

11. Inclusion of staff and possibly others

Health-staff hands over the treatment plan to the medication carer staff who records it in the patient’s record and notifies the results to the resident’s carer staff

**12. Follow-up**

Medication carer staff records main conclusions, and potential noteworthy points in patient’s residence record

8. Consultation preparation

GP and psychiatrist prepare for the shared consultation by orienting themselves in patient record and pharmacist note

10. Treatment plan

GP and psychiatrist send their own record notes for their respectively treatment plan to the health-staff
